# Use of Animal Models in Studying Roles of Antibodies and Their Secretion Cells in Dengue Vaccine Development

**DOI:** 10.3390/v12111261

**Published:** 2020-11-05

**Authors:** Kulkanya Chokephaibulkit, Yu-Wen Chien, Sazaly AbuBakar, Kovit Pattanapanyasat, Guey Chuen Perng

**Affiliations:** 1 Division of Infectious Diseases, Department of Pediatrics, Faculty of Medicine Siriraj Hospital, Mahidol University, Bangkok 10700, Thailand; kulkanya.cho@mahidol.ac.th; 2 Siriraj Institute of Clinical Research, Faculty of Medicine Siriraj Hospital, Mahidol University, Bangkok 10700, Thailand; 3 Department of Public Health, College of Medicine, National Cheng Kung University, Tainan 701401, Taiwan; yuwenchien@mail.ncku.edu.tw; 4 Department of Occupational and Environmental Medicine, National Cheng Kung University Hospital, College of Medicine, National Cheng Kung University, Tainan 701401, Taiwan; 5 Tropical Infectious Diseases Research and Education Centre (TIDREC), University of Malaya, Kuala Lumpur 50603, Malaysia; sazaly@um.edu.my; 6 Department of Medical Microbiology, Faculty of Medicine, University of Malaya, Kuala Lumpur 50603, Malaysia; 7 Center of Research Excellence for Microparticle and Exosome in Diseases, Department of Research and Development, Faculty of Medicine Siriraj Hospital, Mahidol University, Bangkok 10700, Thailand; kovit.pat@mahidol.ac.th; 8 Department of Microbiology and Immunology, College of Medicine, National Cheng Kung University, Tainan 701401, Taiwan; 9 Institute of Basic Medical Sciences, College of Medicine, National Cheng Kung University, Tainan 701401, Taiwan

**Keywords:** dengue, DHF/DSS, B cells, antibody secretion cells, global health, pathogenesis

## Abstract

The cardinal feature of adaptive immunity is its ability to form memory responses that can be rapidly recalled to contain pathogens upon reencountering. Conferring a robust memory immune response to an infection is a key feature for a successful vaccination program. The plasmablasts are cells that not only can secret non-neutralizing antibodies but also can secrete the specific antibodies essential to neutralize and inactivate the invading pathogens. Dengue has been recognized as one of the most important vector-borne human viral diseases globally. Currently, supportive care with vigilant monitoring is the standard practice since there is as yet no approved therapeutic modality to treat dengue. Even though the approved vaccine has become available, its low efficacy with the potential to cause harm is the major hurdle to promote the widespread usage of the vaccine. Despite the decades of research on dengue, the major challenge in dengue vaccine development is the absence of suitable experimental animal models that reflect the pathological features and clinical symptoms, as seen in humans. Dengue is transmitted by the bite of mosquitoes carrying infectious dengue virus (DENV), which has four distinct serotypes. Recently, cases resulting from unconventional transmission routes, such as blood transfusion, organs as well as stem cells and bone marrow transplantations, and mother-to-infant vertical transmission, have been reported, suggesting an alternate route of DENV transmission exists in nature. This review discusses issues and challenges needing to be resolved to develop an effective dengue vaccine. Development of a robust and reliable dengue animal model that can reflect not only dynamic human clinical symptoms but also can answer around why preexisting neutralizing antibodies do not confer protection upon re-infection and immune protection marker for dengue vaccine efficacy evaluation.

## 1. Background

Historically, dengue has been registered and associated with human beings for more than a thousand years [1]. Consequently, numerous descriptions of the clinical signs of dengue have been given, to name a few, such as break-borne fever, dandy fever, three-day fever, seven-day fever, or giraffe fever [2]. A pattern of recurring dengue outbreak with geographic locales has been noticed in the old days, though sporadically emerging and re-emerging throughout the past. However, recent epidemiological evidence indicates that dengue becomes one of the fastest spreading human diseases in the world. Although initially and mainly centralized in tropical and subtropical zones, dengue is now endemic in over 100 countries, and there are no signs of abating since its surfacing in new areas with sporadic outbreaks constantly being reported. Dengue fever has now been recognized as the most common human viral disease and attributed to many epidemics in many parts of the world, especially in tropical Asia, Africa, and South America [3].

Dengue is mainly transmitted by *Aedes* spp. mosquitoes carrying infectious dengue virus (DENV). Upon infection, various clinical symptoms including asymptomatic, mild undifferentiated fever, classical dengue fever (DF), dengue hemorrhagic fever (DHF), and dengue shock syndrome (DSS) have been well-established [4]. The ratio of asymptomatic to symptomatic infections ranged from 1:1 to 20:1 dependent upon the geographic location, immune naivety of the population, and seasons [5,6,7,8,9]. Evidence from healthy blood donors suggests that asymptomatic carrier can be more efficiently spread the virus [10,11]. If the findings are substantiated in other parts of the geographical locales, one of the critical questions is what can be done if asymptomatic individuals carrying high infectious DENV are identified? Since, currently, there is no antiviral modality treatment available. With this note, emerging drug discovery against DENV infection should be at the top priority agenda in public health settings.

For symptomatic dengue, the majority of DF patients can return to normal conditions within a week, while only a low percentage of the afflicted persons may develop severe dengue, DHF/DSS. DF and DHF presentations bear similarities to other acute febrile illnesses, resulting in difficulty for the diagnosis of severe DHF by medical professionals. One of the salient clinical features that can differentiate DHF from DF is the plasma leakage due to an increase in vascular permeability [12]. The criteria and guidelines for the diagnosis of DF and DHF/DSS have been well-established by WHO since 1997, then revised by WHO South-East Asia Regional Office (SEARO) in 2011 [13]. Although the general symptoms during the febrile phase are very similar between DF and DHF, their clinical presentations are different. There are four major criteria defined typical DHF; high fever, hemorrhagic, and often accompanied by hepatomegaly and circulatory failure. However, encephalopathy, severe hepatitis, respiratory failure, and myocarditis have been reported to be atypical clinical signs [14]. One pf the most serious complications in dengue is DSS, which includes disseminated intravascular coagulation (DIC). If not properly managed, shock can occur and lead to terminal death.

Experimental human studies suggest that pre-exposure to DENV can induce protection as a result of the development of immunity, which was first observed in humans by Siler et al. and Simmons et al. [15,16]. The observation provides hope for a preventive vaccine against dengue, but a protective vaccine remains elusive. There are four distinct dengue serotypes, DENV1, DENV2, DENV3, and DENV4, that are concurrently in circulation in endemic areas frequently [17,18]. Pre-exposure to any of the four DENV serotypes has been assumed to confer long-lasting homotypic immunity [17,19], but cases of homotypic reinfection have been reported [20,21,22,23]. The monotypic immunity often offers limited protection to heterotypic reinfection [19,24]. One of the hindrances in assessing the protective effect is that a measurable parameter addressing the correlate of protection or immune response corresponding with reduced pathology that can be evaluated in clinical trials is not available [25]. Multiple molecular mechanisms accounting for the dynamic clinical presentations of disease seen by physicians may contribute to the pathogenic cause of DHF/DSS [26,27].

## 2. Key Factors Affect the Outcomes of Symptomatic Patients

Dynamic clinical features observed in dengue patients may result from intrinsic and extrinsic factors. Age, ethnic or genetic background, and immune and nutritional status of the individual are intrinsic factors. Country locations, circulated virus strains and multiple infections, population density, and competency of the vector mosquitoes are the extrinsic factors. Generally speaking, people of all ages are susceptible to DENV infection, independent of other factors. In clinical presentations, age has been a major factor for the differential presentations of the dengue patients [14,28,29]. Young children have been noticed likely to present with the maculopapular rash [5], while older children, adolescents, and adults are more likely to display classic dengue [30]. DF is usually a self-limited illness, but occasionally, unusual hemorrhagic events associated with severe muscle and joint pain (break-bone fever) can be seen in some patients. Different territory has been shown to present with differential clinical presentations as well. For example, DHF is the major form of severe dengue primarily occurring in children under 15 years in hyper endemic areas, but in other locations, adults appear to be more likely to develop DHF [31,32,33,34]. The reasons for the regional variation in DHF severity remains unknown. Different viral strains in circulation contributing to the altered disease presentations have been proposed by some researchers [32,35,36], while others favor individual genetic background playing a role [37,38].

In order to get a much more comprehensive understanding of dengue, studying the pathogenic parameters involved with the infected host is necessary. Since dynamic clinical course occurs in affected patients and the clinical manifestations change with time, it is very difficult to obtain one specimen at a time point that can completely demonstrate the whole course of pathological features induced by the virus in humans [39]. An attempt to understand the pathogenesis of dengue during the time when thousands of patients die due to DHF in the Philippines and Thailand was made during the early and late 1950s [40]. Autopsy and biopsy specimens were collected for study of the presence of viral antigen in the spleen, lymph node, liver, bone marrow, and skin [41]. Despite tabulating informative results, a definitive conclusion on the pathogenic mechanisms could not be made from these autopsy samples.

High viremia is one of the major clinical features in acute dengue patients, and yet, the morphology of dengue viral particles in the circulation of the infected patients has been more elusive and difficult to convincingly demonstrate [42], despite a well-studied in vitro [43]. Understanding the morphology of virus particles circulating in the patients’ blood is crucial since this could directly impact the efficacy and capacity of neutralizing antibody, which may also affect the precision of antigen diagnostic tools and the evaluation of vaccine efficacy. Recently, the physical status of actual virus morphology in acute dengue plasma has been reported; a form of DENV vesicles in vivo rather than classical dengue virions was obtained from Vero cell culture [44], and these in vivo viral particles are not easy to neutralize by human antibodies [45].

## 3. Pathology and Pathogenesis of DHF/DSS

Because there are very scanty autopsy data available, the pathogenesis of DHF/DSS has not yet been addressed clearly. Salient features on abnormalities include vascular congestions and dilation with edema and multiple focal hemorrhages in most organs, mild to moderate pleural effusion and ascites, mononuclear cell infiltration of interstitial tissues and alveolar walls of lungs, focal myocardial congestion, and a decrease in mature lymphocytes with the proliferation of mononuclear forms in the germinal centers of lymph follicles [40]. Interestingly, no specific damage to the blood vessels has been observed; neither does evidence for vasculitis [46]. The bone marrow often shows maturation arrest of megakaryocytes, and sometimes there is marked generalized cellular hypoplasia with rapid restoration to normal state after recovering from shock [47]. With the expected viremia in acute DENV infection, recovering DENV or detection of virus materials in autopsy organs are reasonable [48,49,50,51], and focal lesions with various degrees of severity can be registered in liver pathology although could be inconspicuous [52,53].

The vascular congestion, dilation, and increased permeability could lead to extensive edema and hemorrhage that were observed in the gastrointestinal tract, the skin, as well as other tissues, leading to loss of plasma volume associated with electrolyte disturbances [54]. In addition, platelet dysfunction and deficiency probably play a role in the hemorrhages [55]. Other parameters such as endotoxin, poor clot retraction, reduction of blood fibrinogen as well as the exhaustion of steroid reserve may also contribute to the circulatory collapse and shock [56]. Evidence suggests that each of the four DENV subtypes can induce DHF/DSS diseases [12,57]. How do the DENV produce such disease remains to be further delineated. Nevertheless, a current hypothesis is leaning toward immunologic reaction contributing to the pathogenesis of DHF/DSS [25,58].

## 4. Animal Models for DENV Infection and Dengue

As of today, despite robust and intensive investigations on dengue, the immunological response and pathological features of DENV infection have not yet been fully delineated, resulting in a major hurdle to develop effective vaccines and therapeutic modalities for dengue. One of the most important tasks in the understanding of the pathogenesis of dengue and dengue vaccine development is the development of an animal model that can capture the salient features of human dengue. These include the ability to support virus replication, induction of clinical signs, and immune responses that are comparable to human dengue virus infection. An attempt to reproduce the key features of the human dengue disease in animals was undertaken during the early 1920s. All in all, more than 500 different animal species were investigated, but the disease was not replicated in these tested animals [16,17,59,60,61]. However, on a positive note, certain aspects of the clinical presentations, for instance, low levels of viremia, were observed in monkeys, murine, and rodents [17,59,62]. On the downside, the pathogenic features of the virus derived from rodent animals were lost after only a few passages [17,63].

Recent advancements in technology and knowledge on animal biology and the scientific understanding gained on the pathogenic causes of dengue in suitable animal models have been improved [64,65]. However, the DENV replication rate in mice is low and serotype-specific resulting in immune response in mouse models required to be further clarified. In addition, the frequently used immunocompromised mouse models lack the interferon receptors and thus cannot develop full immune response [66]. Currently, the BALB/c, A/J, and AG129 immunocompetent models are available for specific questions to be investigated for DENV infection [66]. For instance, BALB/c mouse models enable one to evaluate the innate immune systems or vaccine/antiviral treatment for DENV infection. The A/J mouse models are similar to the BALB/c models but show better relevance to humans in terms of DENV infection and are also feasible for evaluating the innate responses in a vaccine or antiviral studies. The AG129 models are feasible to be infected by DENV and provide much better information for evaluating innate immunity in vaccine studies [65,67]. One of the downsides in immunocompetent mice is that they do not show the symptoms of DENV infection. However, high-dose inoculation in immunocompetent mice can induce clinical signs, and evaluation of the immune responses in these immunocompetent mice model can improve our understanding of disease severity and the potential mechanisms of disease development of dengue [67].

Immuno-deficient or knockout mouse models reconstituted with human cells or cell lines are available for studies of DENV infection. For example, SCID mice reconstituted with human peripheral blood lymphocytes (SCID-PBL), SCID mice transplanted with human cell lines (SCID cell lines), and NOD/SCID-hu mouse models [67,68,69]. It has been suggested that SCID-PBL models are permissive to DENV infection and are a better model for the evaluation of vaccine and antiviral agents than immunocompetent mouse models, but these mice are not good models to evaluate the innate immune system compared to immunocompetent models. SCID cell line models have wider availability than SCID-PBL models, but the application in DENV infection is limited compared to SCID-PBL models. As of today, NOD/SCID-hu models have been implicated to be more relevant for DENV infection than SCID-PBL models since this model is a great tool to study innate immune response to vaccines and antiviral modalities during DENV infection [68]. Moreover, to improve the understanding of dengue pathogenesis and the immune responses in mouse models, several different mice models have been generated. It has been shown that vascular leakage can be induced by the inoculation of mast cell knock-out C57BL/6 mice with two different serotypes of DENV [70]. NOD/SCID mice lacking T cells, B cells, and natural killer cells are transplanted with human cells, a human-like disease mechanism after infection with DENV can be developed [71]. A human-like disease pattern can be observed in NOD/SCID/IL-2Rcc^−/−^ mouse model transplanted with human cells [72,73]. NOD/SCID mouse models transplanted with human fetal liver cells and thymocytes have shown to improve T-cell function, and the use of antiviral agents in these mice is effective [74,75]. STAT^−/−^ mice are permissive to DENV2 infection and present viruses in plasma, the liver, spleen, and nervous system as well as bleeding and blood vessel leakage [76,77]. However, symptoms of neurological disease are noticed in this mouse model, even though neurological events are a less frequent finding in dengue patients. This model suggests it may not relevant to DENV infection in humans. Recently, it has been demonstrated that the immunocompromised AG129 mouse model is sensitive to DENV2, DENV3, and DENV4 infections, providing a tool to study DENV disease patterns, and as such, this model has been widely applied for the efficacy of antiviral studies [65]. STAT1^−/−^/STAT2^−/−^ and type1 IFN receptor (STAT1^−/−^/AR^−/−^) mice are susceptible to DENV infections [78]. Better immune response in a conditional type 1 IFN receptor knock-out mouse model than in the existing immunocompromised model has been shown, which is expected to be useful for identifying future vaccine candidates [66,79]. In general, finding a suitable animal model to investigate the pathogenic mechanisms that mimic the salient features of human dengue and for evaluation of vaccine efficacy remains a challenge [80]. Consequently, to get a better glimpse of the pathogenesis of DENV infection in human beings, a human challenge model for DENV infection has recently been suggested [81,82]. However, with the recent report that found a history of DENV infection may increase the risk of developing leukemia [83], the utility of the human challenge model may remain to be vehemently debated and exclusively scrutinized before proceeding to the experimental stage [82].

## 5. Dengue Vaccines

**Figure 1:**
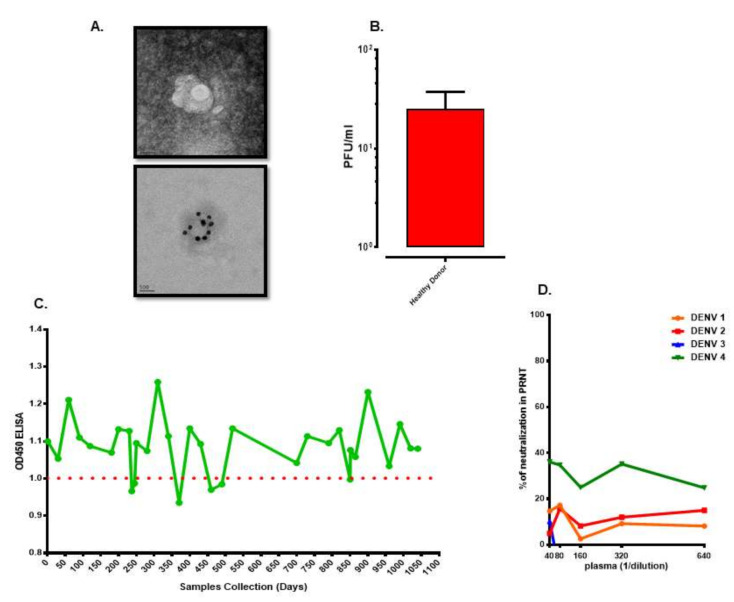
Unique characteristics of viral morphology and antibody capacity in asymptomatic subjects. (**A**) Unique dengue viral morphology. Upper panel was captured by transmission electron microscope (TEM). Lower panel was labeled with dengue virus (DENV) specific E antibody conjugated to 20 nm gold particle and captured by (TEM). (**B**) Viral titer in blood of a healthy asymptomatic subject from dengue endemic country. (**C**) Dengue specific IgG over time in serum of a healthy asymptomatic subject from dengue endemic country. (**D**) Capacity of neutralizing antibody in serum of a healthy asymptomatic subject from dengue endemic country.

Asymptomatic DENV infection accounts for a large portion of affected dengue patients. Transmission of DENV to a recipient infused with blood collected from dengue inapparent subject has been demonstrated since 1939 [84]. Recently, a report shows that asymptomatic humans can efficiently transmit DENV to mosquitoes [10]. Moreover, model analysis suggests that people with asymptomatic DENV infections may account for 84% of DENV transmission [85]. Interestingly, analysis of viral loads in blood donors in Puerto Rico, a dengue-endemic country, revealed that the viral load could be as high as 10^9^ RNA copies/mL of blood in healthy subjects who donated the blood [86]. It is estimated that the incidence rate for daily prevalence in asymptomatic dengue viremia in a city after a major dengue outbreak is on average at 15 per 10,000 people [87]. Blood samples collected from 1391 healthy subjects in a local community at the end of the dengue outbreak revealed that a viral titer can reach 10^5^ PFU/mL of blood in a healthy individual [88]. Since a unique viral morphology, similar to that of cell morphology under EM, in plasma of acute dengue patients has been reported [44]; we submitted the specimen with high viral titer obtained from the asymptomatic subject to EM for viral morphology analysis. The results demonstrated that a unique viral morphology, similar to that of plasma of acute dengue patients, was observed (Figure 1A) in plasma of healthy asymptomatic subject who has very high dengue viral titer. The result indicates that the morphology of DENV circulating in humans is unique and different from that of the tissue culture propagated virus. In addition, infectious DENV could be recovered from a healthy subject originated from the dengue-endemic country, even though the viral titer was low (Figure 1B). Upon following-up on the subject for more than 3 years, serum collected from the subject over time did contain dengue-specific antibody by ELISA (Figure 1C). However, to our surprise, the antibody in this individual did not have neutralization capacity for all four DENV serotypes (Figure 1D). These results suggest that DENV may possess a yet still unknown mechanism to weaken or alter the antibody capacity to react against the virus in a certain subject. Uncovering the mechanisms could benefit from improving the efficacy of the dengue vaccine.

**Figure 2:**
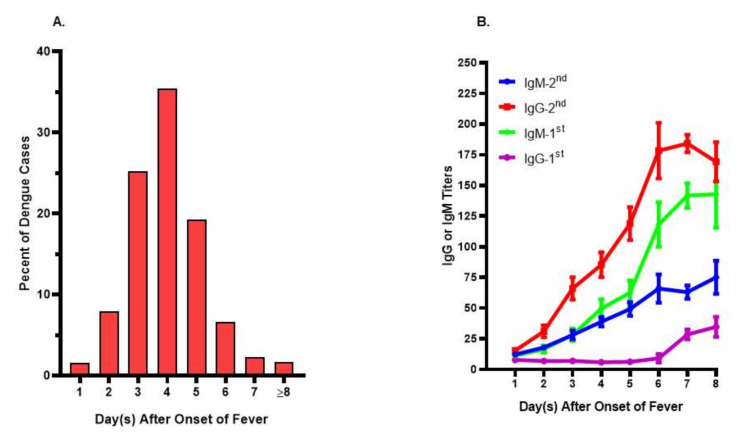
Onset days of fever and immune profiles in acute dengue patients. (**A**) Onset days of fever in dengue patients seeking professional help. The day of fever onset was recorded based upon the recall from dengue patients upon their visiting to the hospital. Patients whose onset day fever was on or after the 8th day were indicated as ≥8. The data were tabulated from more than 1500 dengue patients. (**B**) Immune profiles in patients with primary (1st) or secondary (2nd) DENV infections. The daily IgG or IgM titers during febrile period were measured from sequential specimens collected from 16 primary and 32 secondary dengue patients, respectively. There were low antibody titers during the first couple days of fever in both primary and secondary dengue patients.

As there is no perfect animal model available, it is not an easy task to systematically investigate the immunological factors effectively. Studies have found that the majority of DENV infected subjects would only seek professional help on the fourth day after the onset of fever (Figure 2A). By this stage, there is a 7 to 10 days’ gap from the time of acquiring DENV infection. Despite a well-designed cohort investigation being set up, it is still a challenge to catch an affected subject within the very first days of illness. Consequently, heterogeneous results, some of them even conflicting with each other, have been obtained regarding the pathophysiology and pathogenesis in severe dengue [27,89,90,91,92,93].

Although dengue has been viewed to be a self-limited disease, the complexity of clinical presentations in acute patients has been known and well-documented [12]. During the febrile period, general symptoms in affected patients are fever, malaise, headache, muscle or joint pain, rash, and fatigue. Atypical symptoms such as vasculopathy and coagulopathy as well as platelet dysfunction may be observed in acute febrile subjects [94]. A small percentage of affected subjects may develop severe conditions, which could include rapid plasma leakage leading to circulatory disturbance and bleeding diathesis resulting mainly from thrombocytopenia [94]. As a whole, one of the key features observed in dengue patients is that the illness starts with acute febrile stage, corresponding to the peak of viremia, which could develop into the severe stage of DHF/DSS that occurred at the time of defervescence, corresponding with the clearance of virus in the circulation, and large amounts of immune-related events can be documented. These include overwhelming activation of immune cells [89,95,96,97] and abnormal cytokine profiles [90,98]. Consequently, severe dengue has been viewed to be an immune-mediated disease rather than viral infection illness [25,99], despite reports on the correlation of high viral load during the febrile stage with the progression of severe disease [100].

The salient biological aspect of the adaptive immunity is its recalled capability, existing as a form of memory response that is very rapid and timely to contain the pathogens upon reencountering. Developing a robust memory response to an infection is a crucial feature of the success of the vaccination program in preventive medicine. Scientifically, samples obtained from healthy subjects who have been exposed to DENV infections do demonstrate the recall immunity upon re-stimulation with the viral components [101,102]. Despite evidence suggesting that the magnitude of recall response is critical in controlling the progression of the dengue, and yet, in the case of acute DENV infected subjects, the recall functional immune response seems abnormal [101]. In addition, a low level of memory B cell responses is observed in peripheral blood mononuclear cells collected from symptomatic secondary DENV infection [101]. Moreover, bacterial co-infection infections are not uncommon [103,104], and the clinical similarity between dengue shock and septic shock increases the complexity of treatment in severe cases [105]. These observations suggest that a few scenarios may occur during DENV infection: immunologic non-responding phenomenon [106], the formation of the immune complex [107], viral products induced degradation of existing antibody, DENV-induced defect in the antibody secretion cells, and DENV-induced apoptosis of memory B cells. Why and how these occur remains a mystery.

The best strategy to reduce the dengue burden would be a widespread administration of an effective vaccine. Development of an effective dengue vaccine has been attempted for more than 60 years [3]. Numerous formulations have been tested, including inactivated, attenuated-live, subunits, live chimera-backbone, and DNA-based vaccines [25,108,109,110,111,112,113,114]. Although these vaccines tested in animals and humans showed good immunogenicity with acceptable reactogenicity, there are no FDA approved vaccines available yet for public use until recently. However, the limited efficacy and safety issue of the approved live chimeric tetra-dengue vaccine, Dengvaxia, are major concerns in the widespread usage of the vaccine [115,116]. In the vaccinology term, antibody produces against reinfection is a necessary element to effectively neutralize the incoming intruders. Intriguingly, subjects who are vaccinated 3 times of Dengvaxia^®^ do not appear to be protected, especially in those who were seronegative at baseline, even though mediocre protection has been demonstrated in those who had evidence of previous infection [115,116]. This is a perplexing phenomenon as the vaccine is supposed to induce protective immunity in susceptible seronegative individuals. But lessons learned from the study of Dengvaxia^®^ show that people whose sera are negative for DENV infection are not suitable for Dengvaxia^®^ vaccination. It remains unknown why three doses of Dengvaxia^®^ do not confer a protected effect in these seronegative individuals. One of the major challenging tasks in dengue vaccine trials is that there is no surrogate indicator of immune protection for the evaluation of vaccine efficacy.

Another enigma is around immune non-responding in affected subjects. People living in dengue-endemic regions are more likely to have been exposed to the DENV infection multiple times, and yet, some certain subjects do not have detectable antibodies in their body. This could be because human is one of the natural hosts for the DENV, suggesting that some individuals may not elicit a vigorous immune response to the infection and therefore can cope with DENV, the so-called immune non-responders [11]. Interestingly, a recent report shows that persistent DENV infection can occur in the immunosuppressed patient [117]. Furthermore, literature reports suggest that a possible dengue viral latency may exist. In this particular observation, the author found that even with a single infection, the dengue specific antibody appears to last for more than 20 years [118]. Nevertheless, the site of the possible latency has not been suggested. These individuals may be the reservoir for the mosquito to disseminate the DENV as well as responsible for other routes of viral transmission such as through stem cells and organ transplantations, blood donation, and mother-to-infant transmission [86,119,120,121,122,123]. The recent findings showing that human hematopoietic stem cells are permissive to DENV infection [124], suggest that asymptomatic DENV carriers, persistent and/or latent DENV infection in individuals may present in the natural environment. It would be very interesting to identify these people and to investigate their immune capacity in response to the DENV. The immunity knowledge gained from the asymptomatic subjects, especially on the roles of humoral and cellular immune responses upon DENV infection, may solve a critical issue in developing an effective dengue vaccine.

## 6. Immunity in Dengue Virus Infection

Although recall ability in the immune response is very critical in the development of effective vaccines against many pathogens, the scenario seems not to be that favorable in DENV infection [97,125]. The ability to produce antibody in response to DENV infection appears to wane over time, and oddly, the waning antibody level has been implicated in causing severe dengue upon subsequent infection with other serotypes of DENV [126]. It is a general dogma that cross-reacting antibody with sub-neutralizing ability seems to enhance the progression of the diseases by enhancing viral load upon re-encountering with the DENV, the so-called antibody-dependent enhancement hypothesis [127].

One of the key clinical aspects of DENV infection is that high viremia is observed in patients during the acute febrile stage. Oddly, a limited antibody response occurs during the high viremic period in re-infected subjects that have been well-documented [100,128]. Indeed, our pilot cohort investigation on subjects whose antibody status was based upon the criteria defined by WHO showed that the antibody profiles between primary and secondary DENV infected people were very similar during the first few days of the febrile stage (Figure 2B) The reason is unknown and has not been explored further either. One of the likely scenarios would be that DENV or products released from infected cells (bystander effect) may have a capacity to degrade pre-existing antibody, alter or handcuff the ability of the antibody secretion cells, plasma or plasmablasts.

**Figure 3:**
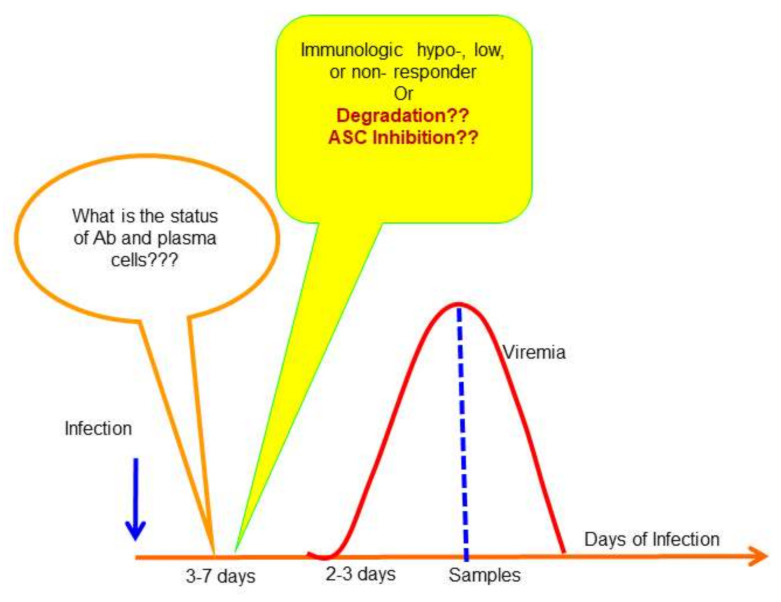
Status of antibody secretion cells and antibodies prior to onset of fever in dengue patients. The diagram described the course of DENV infection in patients. There is a gap about 3–7 days prior to fever onset in affected people. The status of antibody secretion cells is unknown, and the levels of antibodies in those confirmed with previously DENV infected subjects also remain largely unexplored due to lack of a suitable animal model that can reproduce the cardinal feature of human dengue.

A schematic illustration demonstrating the gap that requires researchers’ attention is shown in Figure 3. A major limitation for understanding DENV-specific immune responses is associated with the study design in that samples used for studies are taken from patients in the acute phase of their illness through their recovery period rather than samples obtained during the incubation period, the period between infection and symptom onset). More importantly, most studies did not include asymptomatic or persistent DENV infections. Moreover, the previous studies did not address the role of cellular immunity in early immune protection; rather, they emphasized a correlation between anti-dengue antibody with disease severity. An unbiased natural infection model that includes demonstrable neutralizing antibodies in samples from both asymptomatic and symptomatic infections is needed.

## 7. Conclusions

To better understand the roles of antibodies and their secreting cells, it is imperative to have appropriate animal models to work with. One of the major challenges in dengue research is to have an animal model that can recapitulate the cardinal features of human dengue. Even though there are many approaches to develop the aforementioned animal models, the major obstacle remains in to define the outcomes of the disease and the immune response reproducible in the animal model, and whether it resembles that observed in humans.

With the advancement of technology and knowledge in dengue, the new concept in system biology and epigenetic research, the much more comprehensive picture on the development of a perfect animal model mimicking human dengue would unravel soon. This would lead to the knowledge necessary for vaccine and therapeutic development.

Ethics approval and consent to participate: This studies in Figure 1 and Figure 2 were approved by the institutional review board of National Cheng Kung University Hospital (approval no. A-ER-104-386 and B-ER-104-178). The study objectives and procedures were explained to the participants before obtaining written consent.

## Authors Contributions

Conceptualization, K.C.; writing—original draft preparation, Y.-W.C. and G.C.P.; writing—review and editing, S.A. and G.C.P.; visualization, K.P.; funding acquisition, G.C.P. All authors have read and agreed to the published version of the manuscript.
